# Unusual Location of Residual Mass in an Uncorrected Undescended Testis with Germ Cell Tumor

**DOI:** 10.1155/2023/6626799

**Published:** 2023-10-03

**Authors:** Navid Masoumi, Mostafa Farajpour, Atoosa Gharib

**Affiliations:** ^1^Department of Urology, Shahid Modarres Hospital, Shahid Beheshti University of Medical Sciences, Tehran, Iran; ^2^Department of Pathology, Shahid Modarres Hospital, Shahid Beheshti University of Medical Sciences, Tehran, Iran

## Abstract

Intra-abdominal cancer in an adult with undescended testis (UDT) is rare owing to widespread screening and management during childhood. Here, we present a 33-year-old gentleman with a complaint of abdominal pain and pelvic mass which appeared to be an uncorrected UDT with cancerous degeneration. Additional evaluation revealed a suspicious mass in the retrovesical space which remained after chemotherapy. Histologic evaluation after resection of this mass indicated cancer spread of testicular origin behind the bladder.

## 1. Introduction

Undescended testis (UDT) occurs in about 1% of the male population [1], 5% of which is estimated to be intra-abdominal [2]. UDT is associated with an increased risk of cancerous degeneration, and given that nearly all cases of UDT are diagnosed at an early age, the chance of finding cancer in an abnormally located testis is very slim, as evidenced by the low number of reported cases of cancer in the abdominal testis [3]. The congenital origin of the testes dictates the location of affected lymph nodes in the retroperitoneum, and no aberrant location of lymph nodes or residual masses has been reported before in the absence of scrotal violation [3].

Here, we present the case of an uncorrected UDT with cancer and an unusually located residual mass in the pelvis without any history of intervention for the correction of the UDT in his childhood.

## 2. Case Presentation

A 33-year-old male patient presented with vague abdominal discomfort in the left lower quadrant and a history of infertility. Given his background of a neglected undescended left testicle, an ultrasound (US) was performed which revealed a 2-centimeter mass in the pelvic area. The mass was subsequently removed by a general surgeon (lower midline incision) which was reported to be a mixed germ cell tumor (seminoma and choriocarcinoma). With this finding, he was referred to our urology clinic.

**Figure 1:**
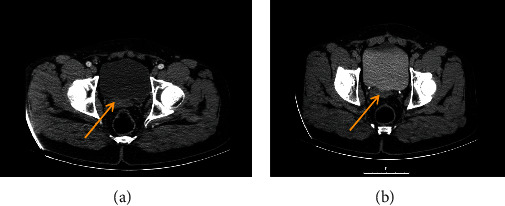
Retrovesical mass, imaging taken after orchiectomy and before chemotherapy.

In physical exam, he was a phenotypically normal male. The chest and abdominal exams were unremarkable, aside from the lower midline incision scar. He had a normal-appearing penis and a right-side testicle. Lab evaluation, two weeks after surgery, revealed elevated *β*-HCG and LDH levels (*β*-HCG: 4645 IU/L (normal range: <2), LDH: 527 U/L (normal range: 105-333)) with normal AFP level (2.34 ng/mL (normal range: 0-40). In computed tomography (CT) imaging, a 40 mm retrovesical mass was detected (Figure 1). Chest CT imaging was negative for metastasis. A tumor marker recheck two weeks later did not indicate any dramatic change. He was also azoospermic in semen analysis.

As of stage III testicular cancer, he underwent BEP ×4 (bleomycin, etoposide, and cisplatin) chemotherapy regimen. Upon completion, the size of the retrovesical mass did not change; however, the tumor marker levels dropped while only LDH remained elevated (*β*-HCG: <0/1 IU/L, LDH: 636 U/L, and AFP: 2/99 ng/mL). In fluorodeoxyglucose–positron emission tomography (FDG-PET) evaluation, the mass did not show any metabolic activity.

**Figure 2:**
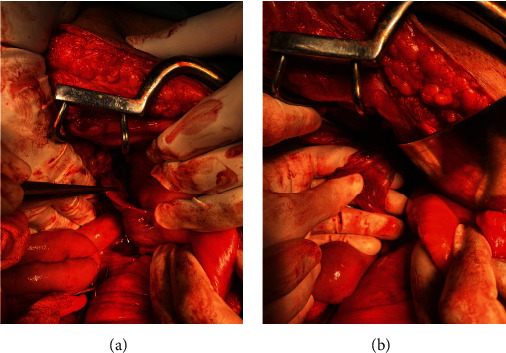
Uterine-like structure located at the dome of the bladder, no ovaries were seen upon further dissection.

**Figure 3:**
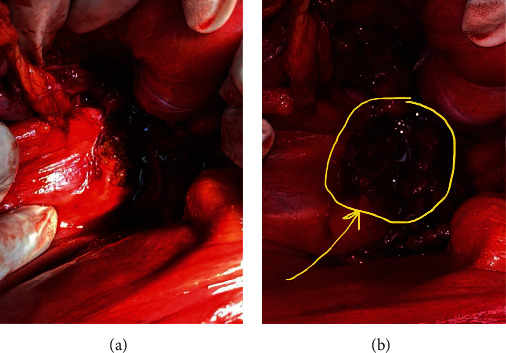
Necrotic tissue behind the bladder at the level of the bladder neck which was scraped off the adjoining structure with wire loop.

With the impression of postchemo residual mass, he was scheduled for nerve-sparing retroperitoneal lymph node dissection (RPLND) with mass resection. Upon entering the abdomen through the midline laparotomy incision, a pear-shaped structure was encountered on the dome of the bladder (Figure 2) which was resected. We continued the dissection behind the bladder, and we encountered grayish necrotic tissue which we had to scrape off from the joining structures (Figure 3). The rest of the surgery was without any difficulty.

**Figure 4:**
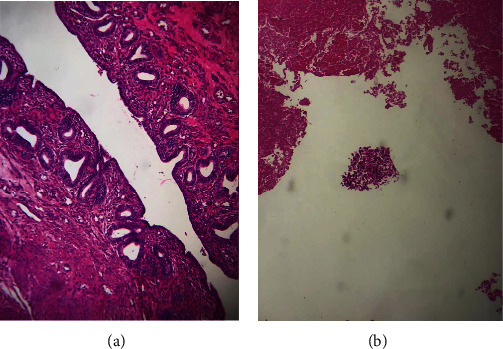
Endometrial tissue (a) and necrotic material with a free-floating cluster of malignant cells (b).

Histologically, the pear-shaped structure was an actual uterine corpus (including endometrium) with a fragment of epididymis and vas deferens. All resected retroperitoneal lymph nodes were benign reactive nodes (53 in total), and the retrovesical tissue was a necrotic material with rare [1, 2] small free-floating clusters of atypical cells (less than 1% of the mass) compatible with minimal residual tumor (Figure 4).

Follow-up karyotyping upon revelation of the uterine structure was 46XY. He was scheduled for regular follow-up. Sixteen months after surgery, tumor marker level and cross-sectional imaging did not reveal any signs of relapse. He was still azoospermic and was sent to a fertility clinic for further treatments.

## 3. Discussion

Undescended testis increases the chance of cancerous degeneration 4 to 6 times in the 3^rd^ to 4^th^ decade of life, but the risk plummets to 2 to 3 times if UDT correction is performed before puberty [4]. Owing to widespread adherence to guidelines regarding early correction of UDT, the number of neglected intra-abdominal testes is very scarce. Less than 15 cases of cancer in abdominal testes have been reported in the literature [1, 3–5], the last of which was from Tunisia in 2022 [4]. However, none of the cases had the unusual location of residual mass encountered in our patient.

As in the previous history of neglected UDT, the possible identity of the pelvic mass was not a difficult guess; however, the unusual location of the retrovesical mass baffled us about its identity. With further evaluation, elevated tumor marker level convinced us of its origin, and chemotherapy was started. A possible reason for this unusual location is the existence of uterus in the pelvis which may have changed the flow of lymphatic tissue. Upon revelation of uterine structure in the pelvis and the history of infertility, karyotyping was performed, and it showed a normal 46XY genotype.

Given the absence of standard management for cancer in intra-abdominal testes, we followed the available guidelines for testicular cancer [3]. Abdominal pain or mass in any patient with a history of uncorrected UDT should prompt the physician to search for cancerous degeneration of the remaining testicle, and uncorrected testes diagnosed at any age should be readily relocated or removed [3].

## 4. Conclusion

In patients with intra-abdominal cancerous UDT, any midline tumors found in the retroperitoneal space should be regarded and treated as a residual mass, and since the literature is poor in this regard, the guidelines on the management of testicular cancer should be followed.
